# Evaluating Transcription Factor Activity Changes by Scoring Unexplained Target Genes in Expression Data

**DOI:** 10.1371/journal.pone.0164513

**Published:** 2016-10-10

**Authors:** Evi Berchtold, Gergely Csaba, Ralf Zimmer

**Affiliations:** Institut für Informatik, Ludwig-Maximilians-Universität München, Amalienstraße 17, 80333 München, Germany; International University College of Turin, ITALY

## Abstract

Several methods predict activity changes of transcription factors (TFs) from a given regulatory network and measured expression data. But available gene regulatory networks are incomplete and contain many condition-dependent regulations that are not relevant for the specific expression measurement. It is not known which combination of active TFs is needed to cause a change in the expression of a target gene. A method to systematically evaluate the inferred activity changes is missing. We present such an evaluation strategy that indicates for how many target genes the observed expression changes can be explained by a given set of active TFs. To overcome the problem that the exact combination of active TFs needed to activate a gene is typically not known, we assume a gene to be explained if there exists *any* combination for which the predicted active TFs can possibly explain the observed change of the gene. We introduce the *i-score* (inconsistency score), which quantifies how many genes could not be explained by the set of activity changes of TFs. We observe that, even for these minimal requirements, published methods yield many unexplained target genes, i.e. large *i-scores*. This holds for all methods and all expression datasets we evaluated. We provide new optimization methods to calculate the best possible (minimal) *i-score* given the network and measured expression data. The evaluation of this optimized *i-score* on a large data compendium yields many unexplained target genes for almost every case. This indicates that currently available regulatory networks are still far from being complete. Both the presented Act-SAT and Act-A* methods produce optimal sets of TF activity changes, which can be used to investigate the difficult interplay of expression and network data. A web server and a command line tool to calculate our *i-score* and to find the active TFs associated with the minimal *i-score* is available from https://services.bio.ifi.lmu.de/i-score.

## Introduction

The goal of many high-throughput experiments is to derive models of regulatory mechanisms that explain the observed changes. For microarray and RNAseq measurements the differential activation of transcription factors (TFs) can be considered a major cause for the observed differential expression of the measured genes.

While there are established methods to measure the mRNA level there is no experimental high-throughput method that can determine which TFs are active, i.e. actually regulate their target genes in the current context. Two experimental approaches are used to infer such activities: ChIP and perturbation (knock-out/-down) experiments. ChIP experiments can, for one TF at a time, determine the binding sites of a TF, whereas KO experiments measure affected genes following an elimination, deactivation, or perturbation of one or several TFs. For the purpose of deriving TF activity changes these experimental methods have a number of disadvantages: (i) binding of a TF to the promoter of a gene alone is not always enough to regulate the gene, as post-translational modifications may be needed to activate the bound TF. (ii) Even if the binding of the TF has an effect on the target gene, it is not clear whether the expression will be up- or downregulated by the bound TF. (iii) Multiple TFs can regulate a gene and it is often unknown whether and how they have to interact to effect gene expression. It is also unclear what the overall effect is if some TFs are activating and others inhibiting. (iv) ChIP experiments are usually not available for all TFs for the experimental conditions analyzed. (v) They are rarely done differentially between two conditions to compare whether the binding of a TF changes. (vi) It is unclear whether the observed changes are a direct consequence of the knocked-out TF or of some downstream regulatory cascade or due to of a side effect of the perturbation itself.

Both types of experiments are context-specific both with respect to the specific bindings and in particular to the effects on possible target genes. Instead of doing differential experiments to uncover which TFs bind in the specific setting, experiments in many conditions can be used to compile gene regulatory networks that indicate which targets may be regulated by which TF in at least one condition. If both knock-out and ChIP experiments are available it is additionally possible to assign a sign to the regulation, that is whether the target gene is up- or downregulated by the TF. Again, the regulation and its sign are likely context-specific.

A goal of many experiments is to uncover regulatory mechanisms that explain the observed differences between the analyzed conditions. Active TFs are the first regulative layer of gene expression and, as the results of the differential activation (i.e. the transcriptional gene expression) are measured directly, can be analyzed more easily than other regulatory mechanisms.

To analyze which TFs are differentially active, computational methods are needed that predict from gene regulatory networks and (a set of) transcriptional measurements the actual activity changes of relevant TFs. This task is challenging as the networks are not complete and, on the other hand, contain many condition-dependent regulations. Furthermore, if a TF is differentially active it does not necessarily regulate all its target genes. Some genes only change their expression if several TFs are active and interact. The precise combination of TFs that have to be active to change the target gene’s expression is rarely known.

Nevertheless, there are several methods which predict the activity changes of TFs or activities from which the changes can easily be calculated [1–3].

Bussemaker [4] introduced a method that models gene expression as an additive combination of TF activities. A matrix *A* that contains the gene expression of several conditions and a matrix *F* that gives the effect strength for each TF-target combination are employed. Multivariate linear regression is used to infer the activities of the TFs *T* via the equation *A* = *C* + *FT*, where *C* is a constant matrix. There are several variants of this approach that differ in the way the TF activities are derived and whether motif occurrence and/or ChIP data is used for the effect strengths of the TFs in *F*. We focus here on *ISMARA* [5] and *plsgenomics* [6] as they are available for assessment as a webserver or as R-package, respectively. The R-package *plsgenomics* takes ChIP data or binary network information for *F* and uses partial least square regression to infer the TF activities. *ISMARA* takes motif occurrences for *F* and is available as a webserver. In addition to solving the undisturbed model, it also solves an *in-silico* knockout model for each TF by removing all regulations of the TF from *F*. The difference between the normal and the knockout model is used to calculate a z-score that indicates how important the respective TF is for the experiments.

*T-profiler* [7] performs a t-test between the fold changes of the genes that are targets of a TF and all other genes. To account for overlaps in the target sets of the TFs, the method iteratively selects the TF with the best p-value and then subtracts the mean expression of the genes in the target set from all genes in this target set. An advantage of T-profiler is that it only needs a gene regulatory network and one dataset of fold changes as compared to the many conditions needed for other methods.

*DREM* [8, 9] uses time series expression data and ChIP data to cluster genes to bifurcating paths of expression changes over time. A bifurcating path indicates that the genes had similar expression values up to this point but diverge systematically afterwards. These bifurcations are then explained by active TFs.

The assessment of the performance of these methods is *generally* very difficult as no gold standard for neither networks nor data nor true activities for TFs are available. To nevertheless systematically evaluate such methods we propose an evaluation score, called the inconsistency score (*i-score*), which indicates, for a given regulatory network, how well the observed changes of the target genes can be explained by a given prediction of differentially active TFs. More specifically it measures the weighted number of gene expression changes, which can **not** be explained by the set of activity changes of TFs in question. This *i-score* is easy to interpret and as it is not optimized directly by any of the methods the *i-score* is well suited to compare and assess their predictions. To compute the *i-score* only a list of TF activity changes, fold changes and the gene regulatory network are needed as input. Thus, results of all mentioned methods can easily be evaluated.

In addition, we provide two methods to obtain the set of differentially active TFs that achieves the best *i-score*. The first method Act-SAT is based on a max-SAT solver and computes the globally best set of activity changes. The second method Act-A* is based on the A* algorithm [10] and computes all optimal solutions which involve only a predefined small number of differentially active TFs. In any case, these optimized *i-scores* constitute the respective theoretical minima given the network and data. These minima can be compared to the *i-scores* of the predictions of various methods to assess how far they are from the optimum. Surprisingly, even if the *i-score* is optimized directly, it is not possible to explain all observed changes. Due to errors in the network or noise in the data many target genes remain unexplained even for the optimal set of activity changes. On the other hand, our Act-SAT and Act-A* methods yield optimal sets of activity changes of TFs explaining most of the observed expression changes. A* delivers such sets with only few differentially active TFs, which are easy to interpret and to use in subsequent analyses and validations. Moreover, the set of unexplained target genes and inconsistent edges might constructively hint to interesting hypotheses implied by the actual data (based on the given regulatory network).

## Material and Methods

### Data and networks

For our evaluation we applied the different methods to three datasets and two networks. Our method can be applied to any organism. In this paper, we focus on yeast as with YEASTRACT [11] a large regulatory network of good quality is available which can serve as a kind of common gold standard for all the methods. The YEASTRACT network comes close to such a standard for yeast, while in most other organisms the situation can be expected to be much worse, i.e. more error-prone, more context-dependent and much more incomplete. In addition to YEASTRACT that contains only experimentally validated regulations, we also include the more putative and much larger motif-derived network used by ISMARA. In this network an edge indicates that the binding motif of a TF matches the promoter of the target. Again, for yeast such a network is more reliable as in other species due to the available data and the assumed complexity of the regulatory mechanisms.

Furthermore, as baseline for comparison, we constructed our own motif-based network using the TF binding motifs provided by Jaspar [12]. We created two different networks by using fimo from the MEME suite [13] (with default parameters, p-value < 10^−4^) to search for binding motifs in the region 250 bp and 1000 bp upstream of the TSS for all yeast genes. The network constructed from the 250 bp upstream of the TSS (called Jaspar 250) contains 40.441 edges and is comparable in size to YEASTRACT (41.498 edges) and the other network (called Jaspar 1000) contains 146.431 edges and its size is comparable to ISMARA (155.404 edges).

As experimental data we used a time series that analyzed the transition of respiratory and respirofermentative cultures to fully fermentative metabolism by monitoring the changes of yeast cultures grown initially with 1% and 20.9% oxygen, respectively, after transition to 0% oxygen [14]. We also used other datasets, not discussed in this paper, and the results are very similar (see S1 File).

Furthermore, to assess the influence of the network systematically we compared the performance for real and randomized data in a large compendium containing many experiments. For this we employed the compendium by Gasch [15] with differential data for 173 experiments measuring the reaction of yeast to several environmental stresses.

### Unexplained target genes

In order to predict the TF activities, the available methods have to make strong assumptions. The regression model used by ISMARA and plsgenomics assumes that the measured expression levels/fold changes are linear combinations of the TF activities. However, it is known that the effects of TFs do not have to be additive and it is possible that a TF can regulate its target gene only if another TF is also active. Thus, if only one of the two TFs is active the expression of the target gene might not change at all. In this case the expression predicted by the regression model will be far from the observed expression as the model is not suitable for this kind of TF-TF interaction, i.e. this particular activation function. For regression models a natural measure to assess the prediction are residuals (the fitting errors per gene). But because of these too restraining assumptions of the model it is not very meaningful to use the residual of the regression fit as a measure of how good the predicted TF activities explain the observed effects. A high residual could either be due to such non-additive effects or due to falsely predicted TF activities.

Here, we propose a more realistic model [16], which needs to be much more general. It is based on Petri nets to model several regulators which could cooperate according to a general activation function to regulate the target gene. This function can depend on binding strength, activity changes, protein concentrations, etc. but is abstracted here as a (maybe complicated) function of the activity changes of the regulating TFs. Such a model may be realistic but, of course, it is not available and cannot directly be used to assess the performance of other (simpler or even more complicated) models. Therefore, rather than formulating a specific model, we introduce the notion of **unexplainability** in order to measure whether activity changes (predictions) cannot explain the data for *any* reasonable activation function. For an explained gene the actual activation function could still be such that the predicted activity changes do not explain the observed effect. Thus, the unexplainability of activity changes of TFs given a regulatory network and data yields a lower bound of the defects (data not explained) of TF activity change predictions. The unexplained activity changes are either wrong, or more and other regulators are required.

As we want to analyze *differential* experiments, we define three activation changes for the TFs. If a TF is similarly active in both conditions we define it to have unchanged activity (*A*^0^). If it is differentially active, it can either be more active (*A*^+^) or less active (*A*^−^). In the following, an active TF always means a differentially active TF.

**Fig 1:**
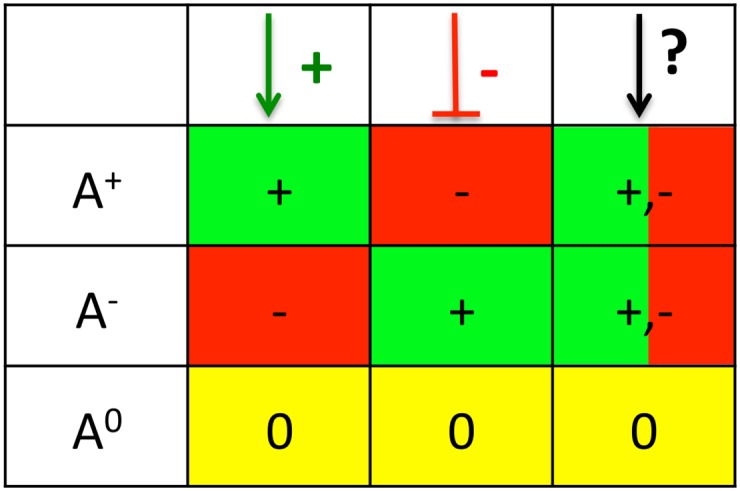
Regulatory effect for all combinations of edge signs (columns) and TF activity changes (rows). E.g. an activating edge (first column) has an activating regulatory effect (+) on the target gene if the associated TF is more active (*A*^+^). If the sign of the edge is not known (last column) the regulatory effect can be assumed to be activating or inhibiting depending on which is needed to explain the target gene.

The **regulatory effect** is the direction of the expected change of the target gene given the annotated sign of the edge (+/-) and the activity change of the TF. A TF can have an activating (+), inhibiting (-) or no (0) regulatory effect on the target gene. Fig 1 shows for all combinations of edge signs and TF activity changes the resulting regulatory effect. If no sign is annotated to the edge, the effect can be either activating or inhibiting, depending on the actual sign of the edge. But as this sign is unknown, we can optimistically assume that the sign is such that the regulatory effect explains the target gene if possible.

A **target gene is unexplained** if for none of its associated TFs the regulatory effect and the target gene’s change are in the same direction.

1. If the target gene is differential (changed) it is sufficient that at least *one* of its TFs is predicted to have the corresponding regulatory effect: as the activation function is unknown, the changing TFs could cause the change of the target (e.g. for an activation function that combines the regulatory effects in an OR-like way).
2. An unchanged target gene is explained if there is at least one TF with no regulatory effect (i.e. one unchanged TF (*A*^0^)). The unchanged TF could be the reason that the target gene is not changed, as the TF is essential for a change (e.g. for an AND-like activation).

Theoretically, it is possible that there are complementary TF activity configurations (i.e. all TFs change their activity) with the same overall effect on the target gene. E.g. if there are two TFs A and B and the target gene is expressed at the same level if either one is active and the other inactive, the target gene can also be unchanged between two conditions if A and B both change but have opposite regulatory effects on the target gene so that they cancel each other out. Strictly, an unchanged target gene could thus only be unexplained if all its TF show the same regulatory effect.

However, we assume that this is rarely the case, as the activity change of both TFs would have to result in the exact same expression change on the target gene so that no expression difference between the two analyzed conditions is observed. Thus, we want to minimize these cases and count the target gene as unexplained if none of its associated TFs are predicted to have unchanged activity (*A*^0^) disregarding complementary TF activity configurations. In addition, we analyzed the effect of these complementary TF activity configurations (see S1 File), but found that the different methods still yield many unexplained target genes.

In the paper we define differential genes by an absolute log fold change above a cutoff *c* of 1, as for some datasets only few replicates are available so that no reliable p-value can be calculated. How p-values can be used and how the results are affected is shown in the S1 File.

### Inconsistency score

If a given prediction of activity changes of TFs is correct one would expect no or only very few unexplained target genes (UTG). The number of unexplained target genes (#UTG) is thus a suitable measure to assess predictions of active TFs. As fold changes of unexplained genes may differ a lot, a weighted inconsistency score might be an even more appropriate measure for the quality of the activity change predictions.

For target genes with a fold change close to the differential cutoff *c* one is less certain whether it is really differential or not, while genes with fold changes far from the cutoff are more certain. This can be taken into account by using a score that incorporates the log fold change of the UTG. The inconsistency score (*i-score*) of a given set of active TFs is calculated as the differences of the absolute log fold change *fc*_*t*_ of the target gene *t* to the cutoff *c*, summed for all UTGs. As differential target genes can have a much larger difference to the cutoff than unchanged genes, the fold changes are trimmed to a maximal log fold change *m*_*fc*_ (default 3).

$$\begin{array}{c} i-\mathrm{score}=\sum_{t\in \text{UTG}}|\min(|f\;c_{t}|,m_{fc})-c| \end{array}$$ (1)

This is of course only one possible way to score the UTGs. One could as well weight differential target genes differently or score only a subset of all genes. The latter may be useful if the user is interested in certain sets of signature genes, pathways and/or GO categories. For our evaluation in the following we use the *i-score* Eq (1) as it constitutes a good balance of the overall inconsistency of all network regulations and the individual differential and unchanged target genes.

### Optimizing the inconsistency score

We applied the *i-score* to the predictions of different methods for several datasets in order to compare their performance. Surprisingly, all methods yield a rather large number of UTGs for most conditions and, thus, a high *i-score*. In order to assess whether the unexplainability is due to the incompleteness and context-specificity of the network or due to noise in the data and not just poor predictions, we calculate the set of active TFs that yields the minimal *i-score*. This minimal *i-score* is the theoretical optimum of the unexplainability given the network and the data.

In the following two optimizations are introduced: one that optimizes the unexplainability without any further restrictions (Activity SAT or Act-SAT) and one that limits the number of active TFs (Act-A*). The second variant is probably more realistic, as one is typically interested in the most important TFs, and a solution in which a large fraction or all TFs are predicted to be active is often meaningless and useless for follow-up experiments. As we employ the optimal *i-score* for assessment and as a comparison of the *i-score* of the actual prediction, even suboptimal scores are useful as lower bounds. Note that even though we optimize the *i-score*, we also report the corresponding #UTG. Both scores together provide a better interpretation as one can assess how many genes are unexplained and how far they are from the fold change cutoff on average.

#### Act-SAT

The optimization of the *i-score* can be modeled as a weighted max-SAT problem [17] and then solved by a weighted max-SAT solver, e.g. akmaxsat [18] or an incomplete weighted max-SAT solver e.g. Dist [19]. SAT is short for Satisfiability which is the NP-hard problem of deciding for a boolean formula whether there is a truth assignment to the variables such that the formula is true. Max-SAT is a variant of SAT where the assignment with the maximal number of satisfied clauses is returned (a clause is the OR combination of several variables, that are combined by AND in the complete formula). While a complete SAT solver guarantees to find the optimal solution, but might take very long, an incomplete SAT solver aborts the optimization after a given time and returns the best solution found so far. Given a SAT formula with weights for each clause, these solvers find the solution with minimal weight for the unfulfilled clauses and, thus, the minimal *i-score*. For our optimization the weighted SAT is given in conjunctive normal form (CNF) as follows. There are three variables for each *TF*_*i*_: one that indicates that the TF is more active ($A_{i}^{+}$), one for less active ($A_{i}^{-}$) and one for a neutral TF ($A_{i}^{0}$). For each target gene *g* one clause is added to the formula:

unchanged target: ${⋁}_{i\in T\;F}A_{i}^{0}$
upregulated target: ${⋁}_{i\in \mathrm{act}}A_{i}^{+}\vee {⋁}_{i\in \mathrm{inhib}}A_{i}^{-}$
downregulated target: ${⋁}_{i\in \mathrm{act}}A_{i}^{-}\vee {⋁}_{i\in \mathrm{inhib}}A_{i}^{+}$

For unchanged target genes the unchanged activity variable of all its associated TFs are combined by OR. For up-/downregulated genes it depends on the sign of the edge which variable is used. For upregulated genes at least one of all TFs with an activating edge to the gene (*act*) has to be more active (*A*^+^) or at least one TF with an inhibiting edge (*inhib*) has to be less active (*A*^−^). Edges for which it is not known whether they are activating or inhibiting are treated as both, so that these TFs are contained in *act* and *inhib*. Note that additional edges (missing in the current network) can only decrease the *i-score* as they imply additional literals in clauses which could satisfy them and thereby reduce the number of unfulfilled clauses.

The weight of each clause is the score that this gene would yield if it is unexplained. The max-SAT solver then finds a solution such that the sum of the weights of the unfulfilled clauses is minimized.

Furthermore, in a valid prediction only one of the three state variables of a TF has to be set to true. Therefore, for each TF the following four clauses are added as *hard* clauses to the SAT formula i.e. they are given a weight that is higher than the sum of all the (soft) target gene clauses. Thus, a solution for which for all TFs exactly one of the state variables is true, always scores better than a solution where not exactly one of the three state variables is true. The first of these clauses guarantees that at least one of the three states is true, and the other clauses guarantee that it is not possible that two states are true at the same time.

$$\begin{array}{c} \left(A^{+}\vee A^{-}\vee A^{0}\right)\wedge \;\left(\neg A^{+}\vee \neg A^{-}\right)\wedge \;\left(\neg A^{+}\vee \neg A^{0}\right)\wedge \;\left(\neg A^{-}\vee \neg A^{0}\right) \end{array}$$

#### Act-A*

Even though SAT solvers are fast for most problem instances it is possible that it takes impractically long to obtain the optimum (the problem is NP hard!). Moreover, if further constraints should be used in the optimization it is usually not straightforward to encode them in the SAT formula. E.g. as it is unlikely that very many TFs are changing their activity it is reasonable to limit the maximal number of (differentially) active TFs, but it is not straightforward to modify the SAT formula to incorporate this constraint.

Therefore, we use a more flexible optimization method based on the A* informed search algorithm [10]. Act-A* iteratively extends partial solutions until all relevant complete solutions are created. It can be used to find the best solution with at most *N* active TFs. A partial solution contains less then *N* active TFs. The search starts with the partial solution with no active TFs, and in each extension step one of the not yet active TFs is set to the more active (*A*^+^) or less active state (*A*^−^).

To enable an informed search by Act-A* we have to estimate partial solutions by an admissible, i.e. optimistic heuristic. In each expansion step the *i-score* of the partial solution is estimated by the admissible heuristic, and if it is worse than the best score of a complete solution already obtained, the partial solution is no longer extended. As the real score of this partial solution is always worse than the heuristic score, optimal solutions are never discarded, but many suboptimal solutions will be skipped.

To calculate the optimistic heuristic score for such a partial solution containing *x* active TFs, we first calculate the (normal) *i-score* of this solution. For each of the not yet set TFs the improvement of this score is calculated, for the two cases where the TF is set to the more (*A*^+^) or less (*A*^−^) active state. These score improvements are sorted and the first *N* − *x* of them are subtracted from the original score. This is an optimistic estimate as the score improvements will decrease with each TF that is set, as target genes are already explained by the set TF and need no longer be explained by other TFs.

## Results

### Active TF predictions are very different across methods

**Fig 2:**
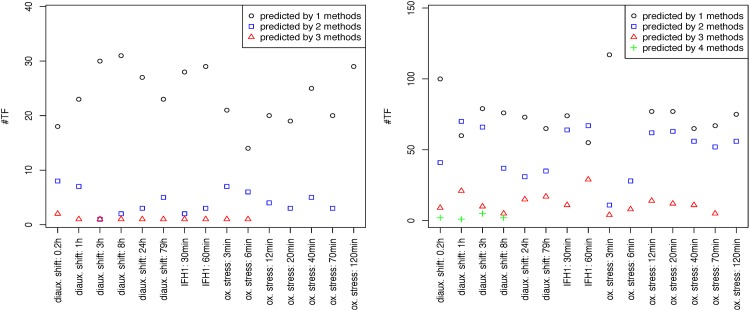
Discrepancy between state-of-the-art methods. For each condition in the selected datasets the number of TFs that were in the top 10 TFs (left) and all predicted TFs (right) for four different methods (ISMARA, plsgenomics, DREM, T-profiler) are plotted. Most TFs are only predicted by one of the methods. There is no TF that was predicted to be active by all methods if only the top 10 TFs were used and only very few if all predicted TFs are considered.

To assess how divergent the predictions of the different methods are, we analyzed how many TFs are predicted by various methods. For each condition of the selected datasets the differential activity of TFs are predicted by all methods and for each TF it is assessed by how many methods it is predicted. This can also be restricted to the most important TFs by restricting the prediction to the top (most changing) 10 TFs. The left part of Fig 2 shows how many TFs are predicted to be in the top 10 TFs by 1, 2, 3 or 4 methods. Surprisingly, there was *no* TF commonly predicted by all 4 methods, only few that are predicted by 3 methods (red triangles) and most TFs are predicted by only one method (black circles). If the unrestricted prediction is assessed (right part of Fig 2) the results are similar and only very few TFs are predicted by all 4 methods.

Thus, the resulting activity changes strongly depend on the used method. Different methods are not even consistent with respect to the most changing TFs. As a consequence, it is especially important to be able to assess which method performs well for a given combination of data and network.

### Performance of methods depends on the particular experiment

**Fig 3:**
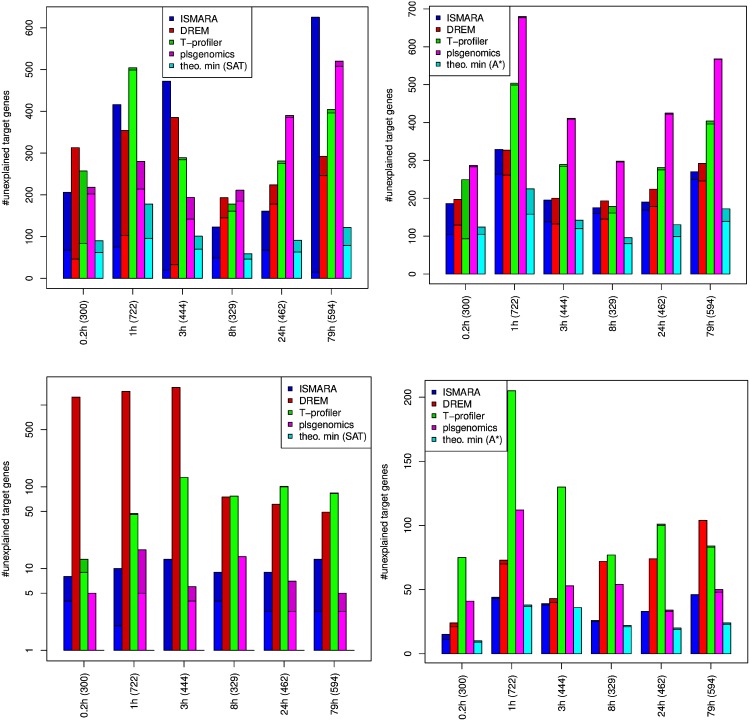
The number of unexplained target genes (#UTG) for the respiratory shift from 20.9%-oxygen time series and the YEASTRACT (top) and ISMARA (bottom) network. On the left all predicted TFs are used and on the right the 10 most important TFs only. The brighter part of the bars indicates how many of the UTGs have changed significantly in the data. The darker part of the bars correspond to the UTGs which are unchanged. For each condition the number of differential genes is given in parentheses.

To assess the different predictions, we calculated the *i-score* and #UTG for the predictions of all methods for the different datasets for both the YEASTRACT and the ISMARA networks. For details on how we derived activity changes from the predictions see the S1 File. The #UTG for the respiratory shift data and the YEASTRACT network are shown in the upper part of Fig 3. For each condition in the dataset the #UTG is given for the different methods. On the left this is shown for the complete prediction while the right plot shows the prediction restricted to the top 10 changing TFs (see S1 File for more details). We also provide the #UTG if the *i-score* is optimized directly, for the unrestricted case on the left by the Act-SAT optimization and on the right by the Act-A* method. The brighter part of the bars indicates how many of the UTGs were differentially expressed.

All methods yielded many UTGs for which the observed effect could not be explained. According to the #UTG, there is no clear best performing method, as these numbers vary considerably across conditions. For the first three timepoints *DREM* and *ISMARA* appear to predict too many active TFs with many targets, as most of the unexplained genes were not significant and there are fewer unexplained significant genes compared to the other methods. This also explains why the scores improve if only the 10 most changing TFs are considered. Only for *plsgenomics* there are considerably more UTGs if only the 10 most changing TFs are considered. This indicates that *plsgenomics* predicts many TFs each of which explains only a small portion of the observed effects. The optimal solutions with respect to the *i-score* for an unlimited number of active TFs (calculated by Act-SAT) and for the 10 most changing TFs (Act-A*) are also comparable so that it appears that 10 active TFs are sufficient to explain the majority of the observed effects for this dataset.

Surprisingly, for the respiratory shift data and the YEASTRACT network there are about 100 target genes that are unexplained even if the *i-score* is optimized. As we make only minimal assumptions and, thus, underestimate the #UTG this is a surprisingly large number. As we underestimate the #UTG a solution with a lower i-score/fewer #UTG does not necessarily have to be better than a solution with a higher i-score/more #UTG. Both are lower bounds which give bounds on the performance of the evaluated methods. The model inherently assumes that the network is true and complete, but current gene regulatory networks contain condition specific edges and are incomplete. If a network contains many incorrect edges solutions yield a good score because these wrong edges are used to explain the effects. As additional edges can only improve the score, UTGs have to be caused by noise in the data or missing edges in the network.

Using the more dense ISMARA network for the prediction and calculation of the *i-score*, the results for the different methods show a larger variation (see bottom row in Fig 3). Especially *DREM* does not seem to be well adapted for such a dense network and predicts too many active TFs. Thus, a huge number of unchanged genes are unexplained as all their associated TFs are predicted to be active and all changing genes are explained. Again, if only the top 10 changing TFs are considered *DREM* performs comparable to the other methods. *T-profiler* also yields many UTGs, but in contrast to *DREM* there are even more UTGs if only the top 10 changing TFs are considered. Almost all UTGs of *T-profiler* were significantly changed in the expression data. Possibly, in the dense ISMARA network the TFs that really regulate these genes are also associated with many unchanged genes so that the t-test is insignificant and the TFs are not predicted to be active. In general, the two regression-based methods *ISMARA* and *plsgenomics* clearly perform better than *DREM* and *T-profiler* if the dense ISMARA network is used.

Overall, when the ISMARA network is used fewer unexplained target genes are observed. However, this does not necessarily mean that the predictions are closer to the truth. The ISMARA network (155.404 edges) is much denser than the YEASTRACT network (41.498 edges). So, the genes are associated with more TFs and it is more likely that at least one activity change is predicted which yields a consistent edge.

### Assessment of networks

To compare the different networks with respect to the *i-score*, we compared the optimal score determined by Act-A* for real and randomized data. For this we shuffled the gene labels of the Gasch compendium [15] 100 times and for each such random dataset calculated the optimal Act-A* solution for all 173 conditions. For each of the conditions, z-scores comparing the *i-score* of the real data compared to the 100 randomized runs are calculated for all networks. Furthermore, we calculated z-scores in the same way for random networks with the same number of edges. To generate the random networks, we kept the TFs and target genes from the original network and added as many random edges as were in the original. Furthermore, we analyzed the effect of the network topology by randomizing the network such that the degree distribution is preserved and calculating z-scores analogously (see S1 File).

**Fig 4:**
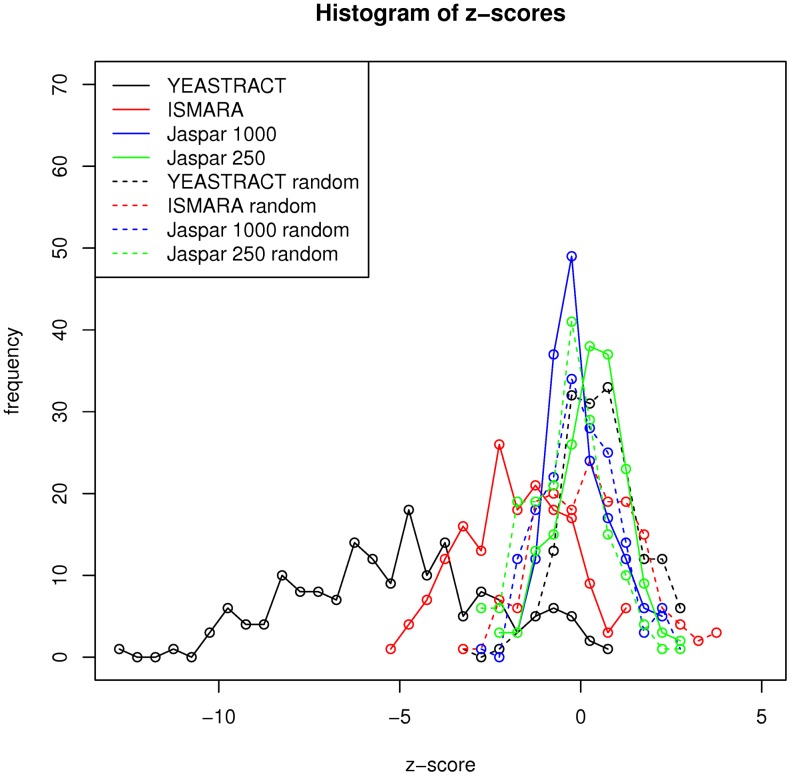
Z-score distributions of the comparison of the *i-scores* of the Act-A* solution for the real and randomized Gasch data. A negative z-score indicates that the *i-score* was smaller/better for the real data than for the randomized data. These z-scores were calculated for the YEASTRACT, ISMARA and Jaspar network and a random network with the same number of edges for each of these networks. When the YEASTRACT network is used the *i-score* is much better for the real than for the random data, whereas the scores are about the same for the Jaspar network.

Fig 4 shows the z-score distributions for the YEASTRACT, ISMARA, Jaspar and the corresponding random networks. A negative z-score indicates that the *i-score* of the real data was smaller than for the randomized data. For the ISMARA and YEASTRACT networks the z-scores for most conditions are negative, while for the Jaspar network and the randomized networks the distribution of the z-scores is centered at 0. For randomized networks there are about equally many unexplained target genes for both the real and randomized data. For both YEASTRACT and ISMARA there is a clear distinction between the z-score distribution for the real and the random network. The network constructed by the Jaspar binding site motifs performs not better than the corresponding random network. The *i-scores* that are calculated using the YEASTRACT network can discriminate better between real and random data as compared to the ISMARA network.

### Variability of solutions

**Fig 5:**
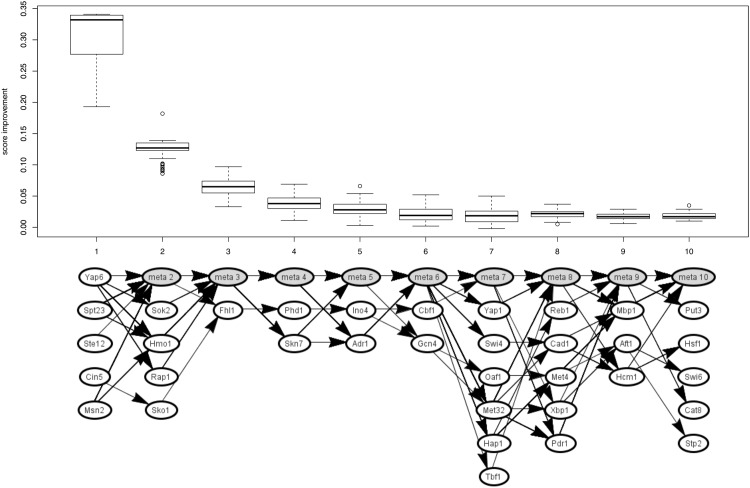
Variability of the 10% best Act-A* solutions. The graph (bottom) shows the active TFs in these solutions. Each path in the graph corresponds to one solution. The position indicates the A* step in which the respective TF is added to the solution. TFs that were added in another solution at an earlier position are collapsed into meta nodes. The boxplot above shows the relative score improvements of the TFs at the given position. Only the 14 shown TFs are used in the first five positions to explain the majority of the effects.

To investigate how much solutions with a good *i-score* differ from each other, we analyzed the best 10% of all solutions scored during the Act-A* optimization. Fig 5 shows boxplots (top) of the obtained *i-scores* improvements and the corresponding solutions (bottom) for the YEASTRACT network and the 1h timepoint of the respiratory shift data. Every solution corresponds to a path in the graph. The position of the active TFs in the path indicates at which step of A* it was added to the solution. If a TF is included in different solutions at different positions only the first is shown and for the other solutions a meta node is introduced at the corresponding position. These meta nodes contain all TFs that were present at an earlier position during the optimization in other solutions. This way, each TF is only present once in the graph at its first position. The boxplot above the graph shows the relative improvement of the *i-score* caused by the addition of the TFs at this position.

The first 5 TFs in any solution explain most of the effects, while the other TFs only explain small fractions of the unexplained target genes (each about 2.5% of the *i-score*). Moreover, there are relatively few alternative TFs used at the first positions, all solutions used some combination of 14 different TFs for the first 5 positions. For the larger positions there are more alternatives that all yield approximately the same (very small) score contribution. So, while the most important TFs are relatively robust across well scoring solutions, the less important TFs are more variable.

### Application to human data

The human gene regulatory network is much larger and more complex than the yeast regulatory network. [20] To demonstrate and evaluate our approach on human data, we constructed a context-specific network from DNase I hypersensitivity and ChIPseq data for two ENCODE cell lines [21, 22] and experimentally validated miRNA-target regulations [23]. The resulting network was used to find the active TFs which yield the minimal *i-score* for the RNAseq data of the corresponding cell lines. Even though the gene regulation is more complex in human and the network might be of a poorer quality, only 261 target genes were inconsistent for 20 active TFs, and many of the active TFs were biologically meaningful (see S1 File for more information).

## Discussion

The prediction of differentially active TFs is an important task for which several tools are available whose performance could so far not be compared systematically. We have shown that the predictions of different methods differ greatly, so that it strongly depends on the used method which TFs are predicted to be differentially active, especially if only the most important TFs are analyzed.

We propose an evaluation strategy to assess how many of the measurements are unexplained for a given set of differentially active TFs. We make minimal assumptions so that we only define a gene to be unexplained if there cannot be a reasonable activation function for the associated TFs such that their activities fit to the measurement. The real activation function, however, is unknown so that it is possible that genes that we assume to be explained are actually unexplained, as the true activation function applied to the predicted active TFs does not result in the observed effect. Thus, the real unexplainability is (grossly) underestimated.

The *i-score* is easy to use and interpret. The number of unexplained target genes (#UTG) is straightforward and gives, especially together with the theoretical minimal #UTG calculated by the Act-SAT or Act-A* method, an intuitive measure of how well the prediction fits to the data. To use the *i-score* only a list of TF activity changes is needed as input (in addition to the data and network that were used for the prediction).

The comparison of the different methods showed that the results strongly depend on the condition as well as the used network so that we could not identify a clear best method. Our analysis did show that not all methods are equally suited for all networks, as some methods are designed to use high quality experimentally derived networks and other methods for more dense (often only inferred) networks. The *i-score* can help to decide which method is best suited for the given network.

Surprisingly, in the evaluation using the YEASTRACT network (which is the current gold standard of yeast gene regulatory networks) many genes were unexplained even for the directly optimized theoretical minimum calculated by Act-SAT or Act-A*. This means that for many genes it is *not* possible to explain the observed effects with the current networks likely because of missing edges in the network. As we make only minimal assumptions and, thus, underestimate the *i-score*, the actual number of unexplained cases will be even higher.

The Act-A* optimization provides the possibility to include prior knowledge. If some TFs are known to be active in the analyzed condition, the Act-A* optimization can be started from the partial solution in which these TFs are set active and then find other active TFs that fit best to the not yet explained effects.

Furthermore, the *i-score* can also be used to explore the effect of individual TFs in a given prediction, by comparing the scores of the solutions where this TF is set to the more (*A*^+^)/less (*A*^−^) active state and inactive (*A*^0^) state, respectively. This way it can be determined whether there is an alternative solution with similar score which does not use the TF in question. Moreover, it allows to add new edges (potential new regulations) or to remove edges and to compute the difference in the i-score. Thus, new regulatory hypotheses can be assessed in the context of the current regulatory network and for the observed data at hand.

## Conclusion

The results of the prediction of differentially active TFs differ greatly between methods and so far there are no systematic approaches and associated evaluation criteria that can be used to assess the performance of different methods. In this study we propose the inconsistency score that evaluates whether given activity changes can explain the measured expression changes. Furthermore, we propose two optimization approaches to determine the theoretical minimum of this score given the data and network. Together, the theoretical optimum and the score for a given prediction are good measures to assess the reliability of the activity changes of TFs and the theoretical optimum can be used to evaluate different networks and to evaluate regulatory hypotheses. Thus, the *i-score* is a useful tool for the analysis of any large-scale dataset.

## Supporting Information

S1 File Supplementary Information. (PDF)
