# Supplementary material for: Evaluating Transcription Factor Activity Changes by Scoring Unexplained Target Genes in Expression Data
Source: PLoS One. 2016 Oct 10;11(10):e0164513. doi: 10.1371/journal.pone.0164513 (PMC5056719; doi:10.1371/journal.pone.0164513)
Supplement: S1 File — (PDF) [file pone.0164513.s001.pdf]

Supplement: Evaluating Transcription Factor Activity Changes by Scoring Unexplained Target Genes in Expression Data

Evi Berchtold, Gergely Csaba and Ralf Zimmer

1 Used networks

The following table summarizes the used yeast gene regulatory networks. It gives the number of TFs, target genes, edges, the mean target in-degree and how many of the edges are annotated with an effect, that is whether the edge is activating or inhibiting.

network	#TFs	#targets	#edges	$\frac{\#edges}{\#targets}$	mean target degree	#edges with effect
Yeastract	172	6370	41498	4.93	7.38	8714
ISMARA	163	5686	155404	27.33	27.33	0
Jaspar 250bp	159	6636	40441	6.09	7.29	0
Jaspar 1000bp	159	6689	146431	21.89	28.68	0

2 Evaluation of activity state predictions

To calculate the inconsistency score we need a list of activity states of TFs. The methods that we want to compare use different inputs and provide different outputs, so that we first have to define which TFs we classify as more and less active for each method.

T-profiler [2] returns a list of TFs that had a p-value below 0.05 in the iterative t-test. These TFs are used as active TFs but we do not know whether they are more or less active.

ISMARA [1] and plsgenomics [3] return an activity matrix with activity values for each TF in each condition. For plsgenomics, we assume a TF to be active if the absolute value of this activity is above some threshold (0.05) and the sign of this value indicates whether the TF is more or less active. ISMARA is available as fully automated webserver to which the user uploads the raw expression data. While this is usually comfortable, it also restricts the possibilities to vary the inputs. ISMARA is not designed to process a user defined network or differential expression values. As we are interested in differential TF activities and apply all other methods to fold change data, we calculate activity differences from the activities returned by ISMARA. If a_1 and a_2 are the estimated activities in the two conditions, and δa_1 and δa_2 are the error bars indicating the uncertainty in the estimates (outputted by ISMARA), then one can calculate the z-statistic

$$z = \frac{(a_1 - a_2)}{\sqrt{\delta a_1^2 + \delta a_2^2}} \tag{1}$$

TFs for which the absolute value of the z is at least 2 are predicted to be active.

DREM [6] returns a graphical representation of the clustered paths and annotates the active TFs to the timepoint at which they were first active. We regard a TF to be active as long as the path annotated with the active TF does not return to the expression level before the split.

T-profiler and DREM do not predict whether the active TFs are more or less active. To nevertheless calculate the inconsistency score for networks with annotated effects for the edges, we optimize

the activity state of the active TFs by counting how many target genes would be explained by the TF if it is more or less active and setting it to the state that yields more explained target genes.

2.1 Comparison to baseline methods

To further evaluate the analyzed methods, we included two baseline methods: (1) the method called *expression* predicts all differentially expressed (|log2 foldchange| > 1.0) TFs to be active. (2) The *enrichment* method which performs a hypergeometric test for each TF comparing target genes of the TF with the differentially expressed genes. TFs for which this test is significant (Bonferroni corrected p-value <0.05) are predicted to be active.

Surprisingly, both baseline methods perform rather well with their #UTG slightly worse or comparable to the other methods, but worse than the i-score/#UTG.

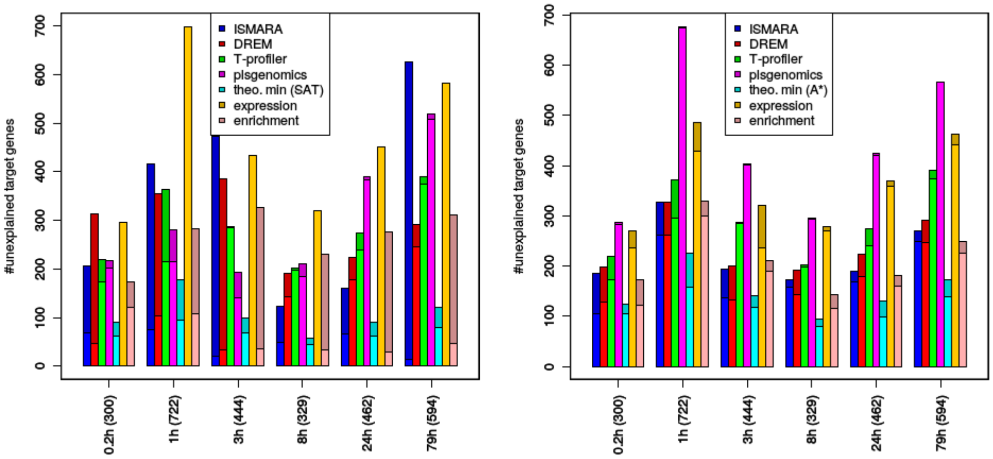

Figure 1: #UTG for the **respiratory shift** time series for the YEASTRACT network including baseline methods. On the left all predicted TFs were used and on the right the prediction was restricted to the 10 most active TFs. The brighter part of the bars indicate how many of the UTGs have changed significantly in the data. For each condition the number of significant genes is given in parentheses. The basline methods perform only slightly worse or even comparable to the other methods.

2.2 Definition of the most important active TFs

All the used methods return some score for each TF that can be used to rank the TFs and select the most important active TFs. T-profiler returns a p-value for each TF so the TFs with the most significant p-values are selected as the most important ones. ISMARA and plsgenomics return z-scores and activity values, respectively, that should be higher for more important TFs. For DREM the active TFs are ordered by the returned p-value at the corresponding split. To get the x most important TFs that optimize the i-score, the A* optimization can be used with x active TFs.

3 Performance comparison for other datasets

The #UTG was also calculated for the predictions of all methods for the oxidative stress [11] and the IFH1 overexpression [10] time series. Figure 2 and 3 show on the top the #UTG for the YEASTRACT [12] and on the bottom for the ISMARA network. On the left the results for the

unrestricted prediction is shown, and on the right if only the top 10 active TFs were used. Similarly to the diauxic shift dataset shown in the paper it depends on the experimental condition which method performs best.

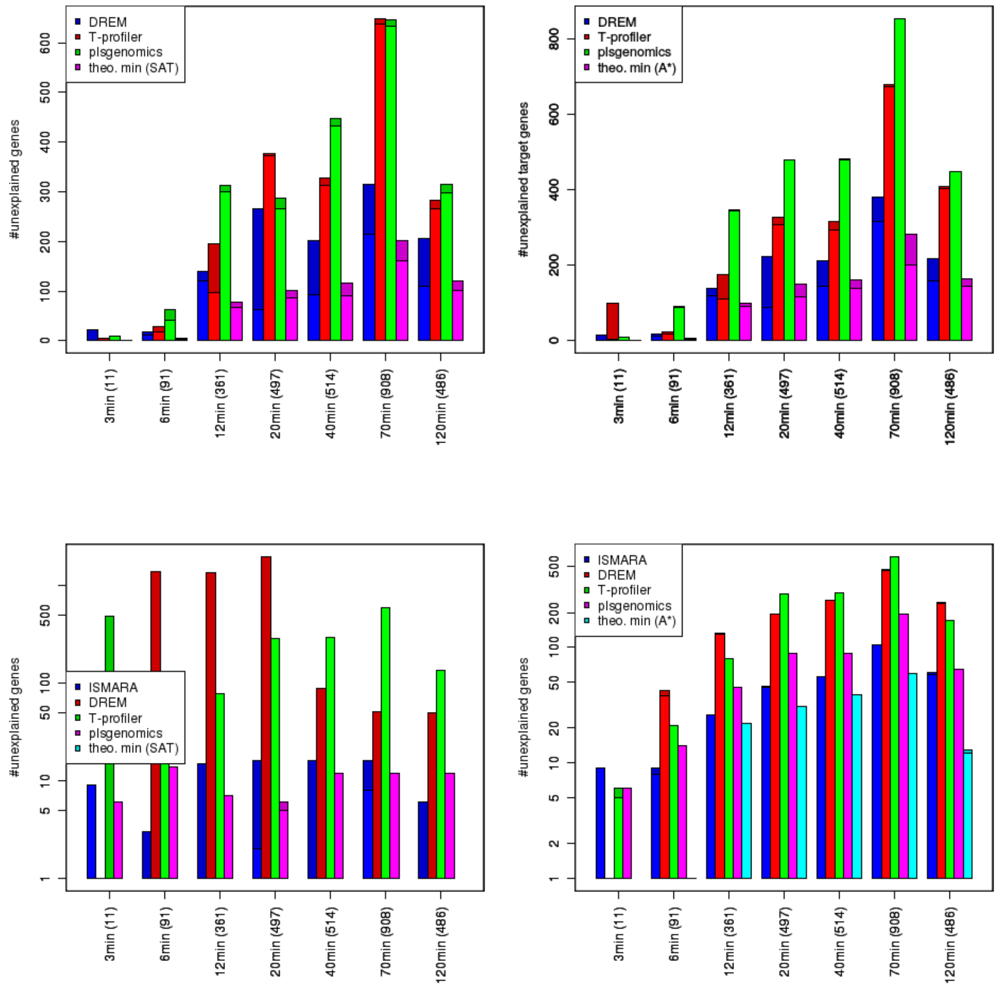

Figure 2: #UTG for the **oxidative stress** time series for the YEASTRACT (top) and ISMARA network (bottom). On the left all predicted TFs were used and on the right the prediction was restricted to the 10 most active TFs. The brighter part of the bars indicate how many of the UTGs have changed significantly in the data. For each condition the number of significant genes is given in parentheses.

4 Definition of differentially expressed target genes

To allow an application for any differential dataset, the i-score uses only foldchanges. However, if enough replicates are available it is better to determine the differential target genes using a statistical test e.g. a t-test. Thus, if both a p-value and foldchange are available, the easiest way to incorporate this in the i-score analysis is to remove target genes for which p-value and foldchange do not agree. If, e.g., the target gene is significantly differential according to the p-value but not according to the

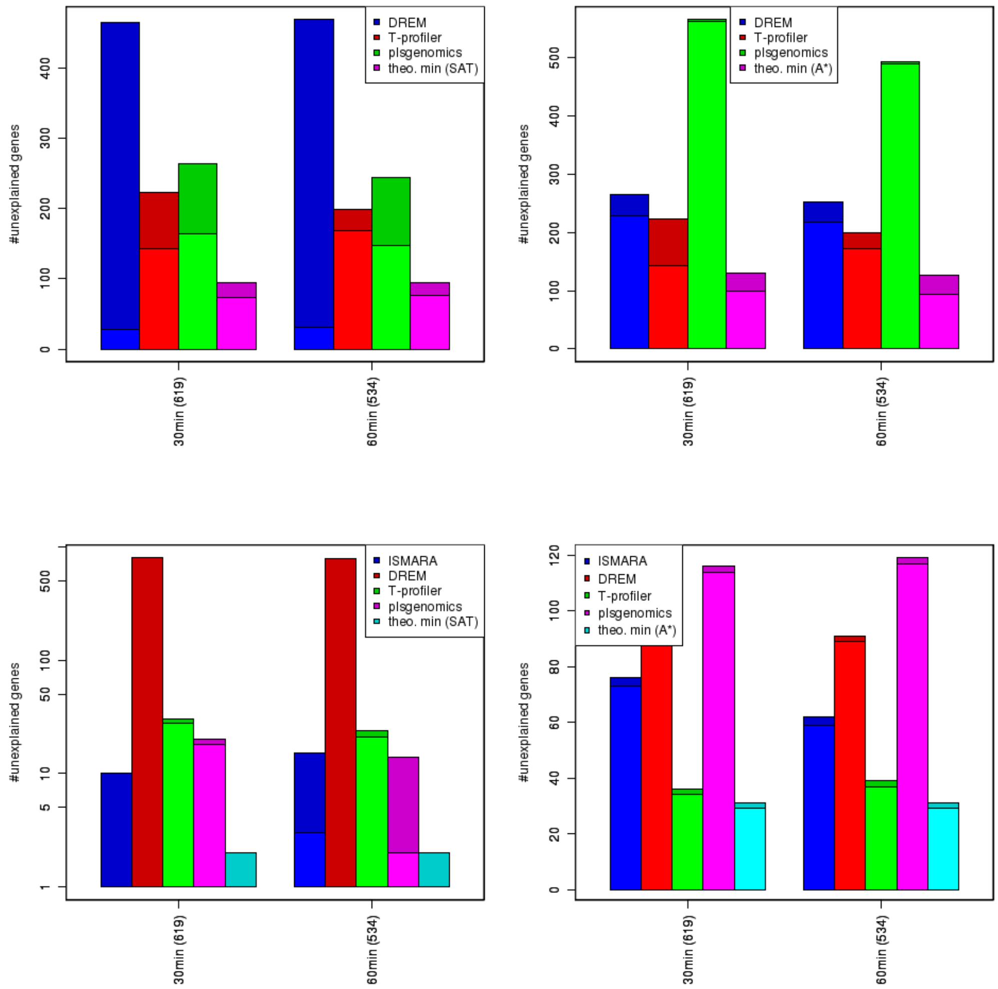

Figure 3: #UTG for the **Ifh1 overexpression** time series for the YEASTRACT (top) and ISMARA network (bottom). On the left all predicted TFs were used and on the right the prediction was restricted to the 10 most active TFs. The brighter part of the bars indicate how many of the UTGs have changed significantly in the data. For each condition the number of significant genes is given in parentheses.

foldchange (only small foldchange), one is not sure whether it is really differential or not and it might be better to omit the target gene altogether from the calculation of the i-score/#UTG.

For the oxidative stress time series three replicates are available for each measurement, so that p-values can be calculated using a t-test with Benjamini-Hochberg correction. Target genes with a p-value below 0.05 and an absolute foldchange below 1, or a p-value above 0.05 and an absolute foldchange above 1 were considered inconsistent and are highlighted in Figure 4. For most methods and time points a larger fraction of the unexplained target genes than expected had inconsistent foldchanges/p-values, so that at least some of the unexplained target genes appear to be due to a wrong classification whether the gene is differentially expressed. However, even if all the target genes with inconsistent foldchange/p-value would be misclassified (which is highly unlikely) all methods still exhibit a quite large number of unexplained target genes.

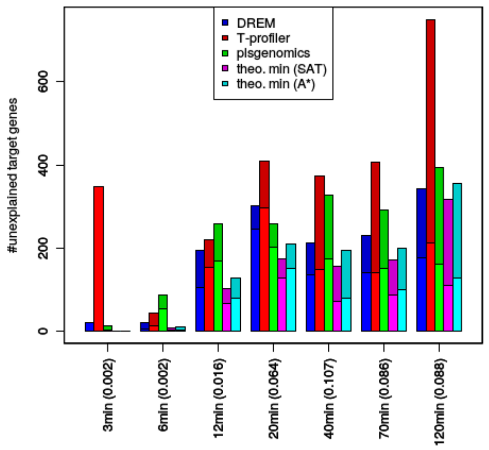

Figure 4: #UTG for the oxidative stress time series and the YEASTRACT network highlighting the inconsistent unexplained target genes. The brighter part of the bar corresponds to the unexplained target genes when target genes with inconsistent p-value and foldchanges are ignored. So the difference between the dark and bright bars is the number of unexplained target genes with inconsistent p-values/foldchanges. The number in parentheses gives the fraction of all target genes with inconsistent p-value and foldchanges.

5 Influence of the foldchange cutoff

We evaluated the influence of the foldchange cutoff on the resulting i-score/#UTG by comparing the results for a cutoff of 1 and 0.7 (see Figure 5). Of course, the performance measures (#UTG and i-score) change if the foldchange cutoff is altered. All target genes that change their differential expression status (i.e. are differential with the new cutoff, but were not differential with the old cutoff, or the other way round) and were unexplained will be explained with the new cutoff. However, some of the former explained target genes that changed their differential expression status will be unexplained with the new cutoff. So the performance measures vary depending on how many target genes change their differential expression status, and the change can be in both directions.

The #UTG are generally higher if an absolute foldchange of 0.7 is used compared to a cutoff of 1, so it seems that 1 is the more suitable cutoff to define the differentially expressed target genes. In any case, the number of #UTG is surprisingly high for both cutoffs.

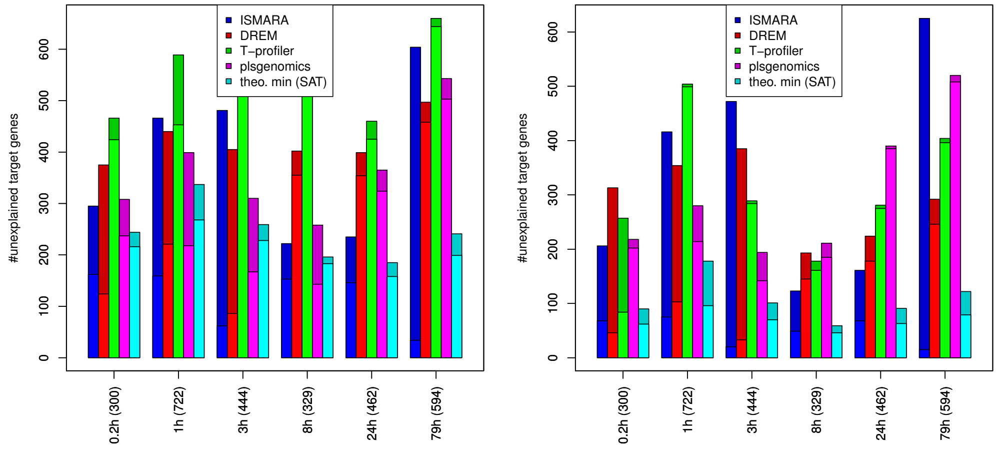

(a) abs. foldchange cutoff: 0.7 (b) abs. foldchange cutoff: 1.0

Figure 5: Influence of the foldchange cutoff on the #UTG. The #UTG differ for the two cutoffs and are generally lower for 1.0.

6 Active TF predictions are inconsistent across methods

For each condition of the selected datasets the activity of TFs are predicted by all methods and for each TF it is assessed by how many methods it is predicted. Figure 6 shows the results of this analysis. Only for some conditions there are TFs that were predicted by all TFs (pink circles). Most TFs are only predicted by one (black) or two (blue) methods.

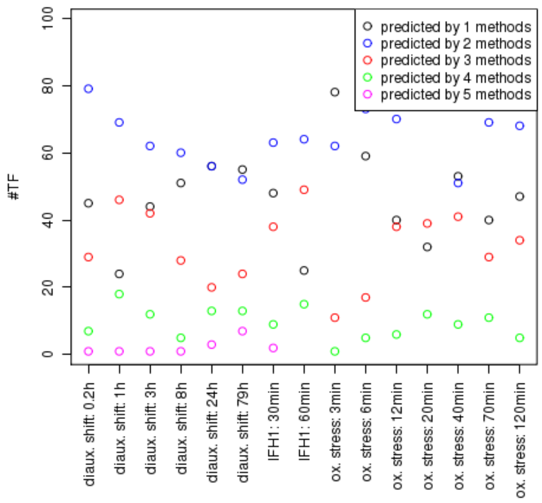

Figure 6: For each condition in the selected datasets the number of TFs that were predicted by multiple methods is plotted. Most TFs are only predicted by one of the methods. There is no TF that was predicted to be active by all methods.

7 Inconsistency scores

Figure 7 shows the i-scores for the predictions using the diauxic shift [9] time series and the YEASTRACT (top) and ISMARA (bottom) network. The results are similar to the #UTG with respect to the ordering of the methods and differ only in the scale.

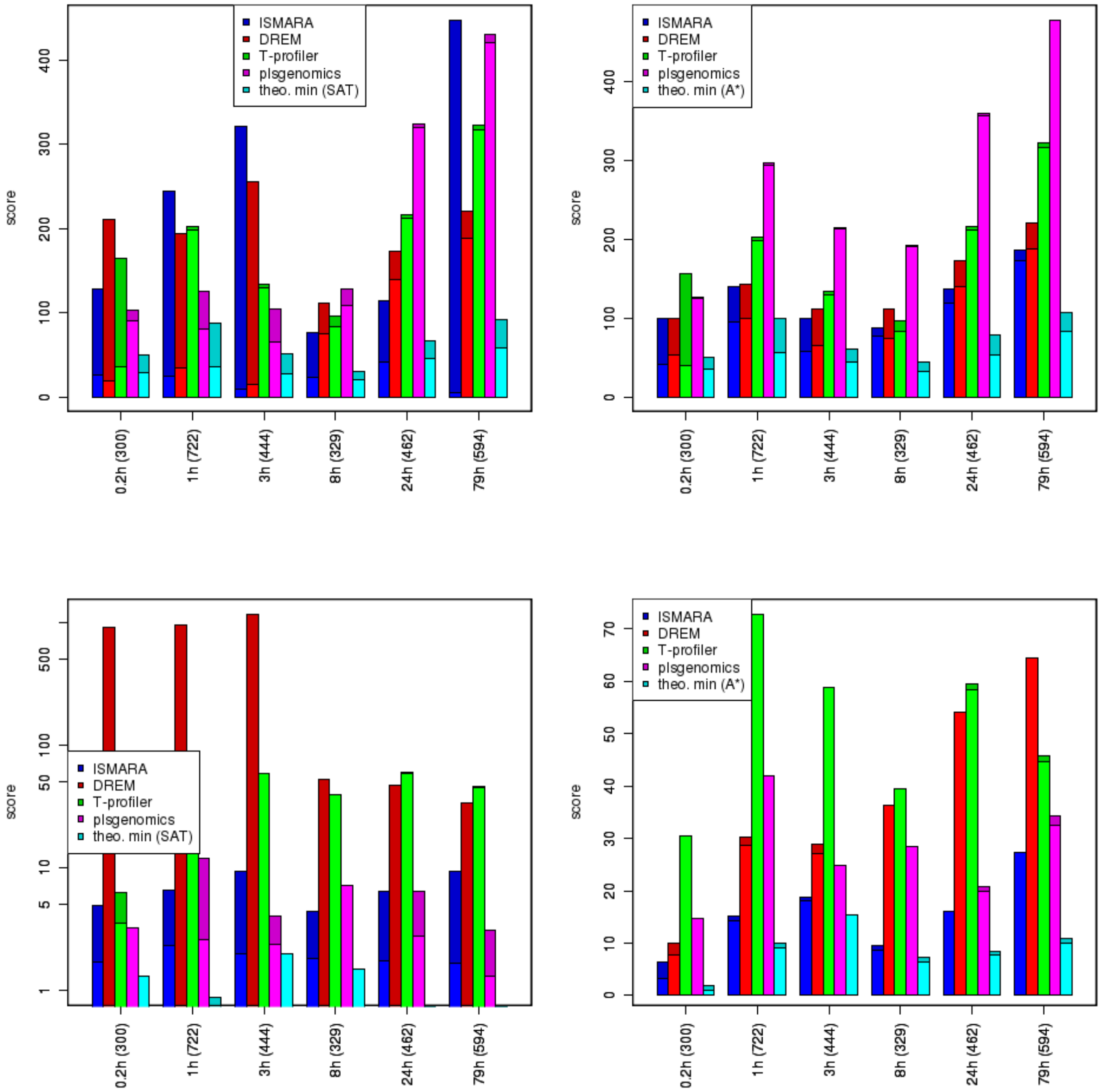

Figure 7: i-scores for the diauxic shift time series and the YEASTRACT (top) and ISMARA network (bottom). On the left all predicted TFs were used and on the right the prediction was restricted to the 10 most active TFs. The brighter part of the bars indicate how many of the UTGs have changed significantly in the data. For each condition the number of significant genes is given in parentheses.

8 Definition of unchanged unexplained target genes

For target genes that do not change their expression between the two compared conditions there are two ways to define when it is unexplained. If one of the associated TFs is predicted to be unchanged (A^0) the target gene is explained in both cases, as the unchanged TF could be essential for a change in the target gene's expression. Even if all TFs are either more (A^+) or less active (A^-) it is theoretically possible that there are two complementary TF configurations (i.e. all TFs are changing between the two conditions) that result in the same target gene expression level (i.e. the target gene does not change between conditions). The most simple example is a target gene with two TFs A and B that yield the same target gene expression level if either one is active and the other inactive. If in one condition A is active and B inactive and in the other condition the other way round, the differential TF activities are A^- for A and A^+ for B so that both TFs are changing even though the target gene is unchanged. In order to not regard these cases as unexplained target genes, an unchanged target gene is only unexplained if all its TFs have the same regulatory effect.

However, we assume that such cases should be very rare as the two complementary TF configurations would have to result in exactly the same target gene expression level. So, for the i-score an unchanged target gene for which all associated TFs are predicted to be changing is unexplained. Nevertheless, we analyzed the number of unexplained target genes using the alternative definition of unexplained target genes that takes complementary TF configurations into account. Figure 8 shows the #UTG for both definitions of unexplained target genes. For plsgenomics we optimize the activity threshold above which a TF is active, so that the set of active TFs is different for the two UTG definitions. The number of unchanged unexplained target genes decreases especially for ISMARA and DREM, but there are still many unexplained target genes for all methods. This indicates that especially the solutions of ISMARA and DREM imply many target genes with complementary TF configurations even though these cases should be rare. This is most likely the effect of too many predicted active TFs as both methods predicted more than 30 active TFs with a marked reduction in the #UTG (this applies to all conditions for ISMARA and the first three timepoints for DREM).

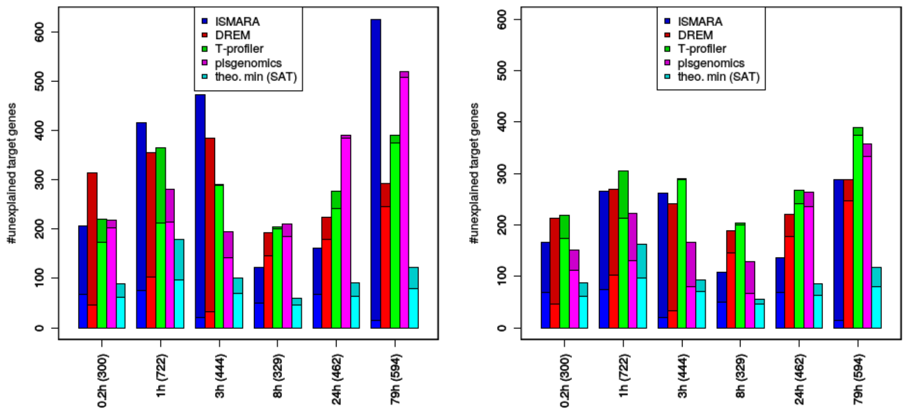

Figure 8: Comparison of the number of unexplained target genes (#UTG) with the normal definition of unexplained genes as used in the paper (left) and the alternative definition that also allows unchanged target genes to be explained if all its TFs are changing (right).

9 Application to human data

9.1 Network

Arguably, in contrast to more primitive model organisms such as yeast, there is no comprehensive gene regulatory network of good quality for human. However, the ENCODE [4] project provides a large number of cell type specific ChIPseq and open chromatin sequencing data that can be used to construct context specific networks for the comparison of two cell lines. To check how our method performs on human data, we applied it to RNAseq data from two cell lines and a network that is as specific for these cell lines as possible.

We selected GM12878 and K562, the two cell lines with the largest number of ChIPseq experiments for TFs and obtained context-specific networks from the CroCo Database [8] and combined them as follows: we constructed a DNase I hypersensitivity sequencing (DNase-seq) network for both cell lines, by searching for motif hits in the DNase-seq peaks in the promoter (+/- 10kb from TSS) region. For those TFs for which ChIPseq data is available, the edges from the DNase-seq based network are removed and replaced by edges derived from the ChIPseq data, as ChIPseq is considered as the more specific measurement. In the ChIPseq experiment it is measured directly whether the TF binds to the given site, while the DNase-seq measurement only indicates that the chromatin is open at this site and that there is a motif hit for the TF.

TFs are not the only regulators of gene expression in higher eukaryotes. Another mechanism is the down-regulation of gene expression by miRNAs. To include this source of differential regulation, we added miRNA-target regulations that were experimentally validated from mirTarBase [7].

The resulting network contains 1.883.156 edges of 321 TFs and 578 miRNAs.

9.2 Expression data

As expression data, we used the ENCODE RNAseq data for the two cell lines, and calculated local fold changes [5] between them. This method returns not only the fold change, but also a p-value that indicates how reliable this fold change is and a credibility interval. We defined a gene to be differentially expressed, if the following holds:

- the absolute log fold change is above 1 for all replicates
- the p-value is below 0.01 for all replicates
- the credibility intervals of all replicates do not contain 0 (i.e. it is likely that the gene is indeed up- or downregulated)

Using this criteria we observed 9.174 differential genes of which 5.864 were target genes in our network.

9.3 Results

We applied our Act-A* method to determine the set of active TFs that yielded the minimal i-score. For this analysis the maximal number of active TFs was set to 20, in order to identify the main regulators and avoid overfitting. The i-score for this data was 427.45 with 261 unexplained genes. Table 1 shows that the vast majority of target genes was consistently explained. Only few target genes could not be explained, and of these there were more unexplained differential genes than unchanged genes. Given the relatively high i-score for this number of unexplained genes, the unexplained genes seem to have high fold changes. This could indicate that more than the 20 selected TFs are differential, so that the selected active TFs are not enough to explain all changing target genes. However, if the number of active TFs is increased, the #UTG and the i-score are increased.

Both cell lines are derived from blood of female donors. The GM12878 cell line was derived by EBV transformation while K562 is derived from a patient with chronic myelogenous leukemia. Several of the predicted active TFs are biologically meaningful as they are associated with virus

	explained	unexplained
differential	5654	210
unchanged	11390	51

Table 1: Number of genes that are explained and unexplained for the predicted 20 active TFs, respectively.

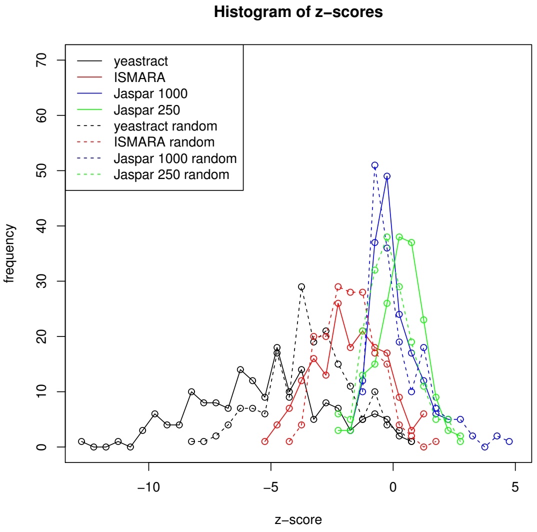

Figure 9: Z-score distributions of the comparison of the i-scores of the Act-A* solution for the real and randomized Gasch data. A negative z-score indicates that the i-score was smaller/better for the real data than for the randomized data. These z-scores were calculated for the YEASTRACT, ISMARA and Jaspar network and a random network with the same degree distribution for each of these networks. When the YEASTRACT network is used the i-score is much better for the real than for the random data, whereas the scores are about the same for the Jaspar and ISMARA network.

infection related processes such as immune response (e.g. CCNT2, IRF3, TRIM28 or USF1) or are associated with cancer related processes such as proliferation and apoptosis (e.g. HDAC2, ETS1 or TAL1).

10 Assessment of networks

In addition to completely random networks, we also assessed the influence of the degree distribution of the network, by randomizing the networks such that degree distribution is preserved. For these random networks, again, a z-score comparing the real and randomized data for each condition in the Gasch compendium is calculated and compared to the z-scores of the real network.

Figure 9 shows these z-score distributions for the real and randomized networks. In contrast to the completely randomized networks, the z-score distributions of the degree-preserved random networks of YEASTRACT and ISMARA are not centered around zero but shifted towards negative z-scores. This means that even randomized networks with this degree distributions are able to explain more target genes in the real data compared to the random data (i.e. they yield smaller i-scores/#UTG for the real data). For YEASTRACT, the z-scores for the real network are still smaller than the z-scores of the randomized network. So, the real data is better explainable using the real network than the randomized network, even if the degree distribution is preserved. On the other hand, for ISMARA the randomized and real networks yield approximately the same z-score distribution.

References

[1] P. J. Balwierz, M. Pachkov, P. Arnold, A. J. Gruber, M. Zavolan, and E. van Nimwegen. ISMARA: automated modeling of genomic signals as a democracy of regulatory motifs. *Genome Research*, 24(5):869–884, 2014.

[2] A. Boorsma, B. C. Foat, D. Vis, F. Klis, and H. J. Bussemaker. T-profiler: scoring the activity of predefined groups of genes using gene expression data. *Nucleic Acids Research*, 33(suppl 2):W592–W595, 2005.

[3] A.-L. Boulesteix and K. Strimmer. Predicting transcription factor activities from combined analysis of microarray and ChIP data: a partial least squares approach. *Theoretical Biology and Medical Modelling*, 2(1):23, 2005.

[4] ENCODE Project Consortium and others. An integrated encyclopedia of dna elements in the human genome. *Nature*, 489(7414):57–74, 2012.

[5] F. Erhard and R. Zimmer. Count ratio model reveals bias affecting NGS fold changes. *Nucleic Acids Research*, 43(20):e136, Nov 2015.

[6] J. Ernst, O. Vainas, C. T. Harbison, I. Simon, and Z. Bar-Joseph. Reconstructing dynamic regulatory maps. *Molecular Systems Biology*, 3(1), 2007.

[7] S.-D. Hsu, Y.-T. Tseng, S. Shrestha, Y.-L. Lin, A. Khaleel, C.-H. Chou, C.-F. Chu, H.-Y. Huang, C.-M. Lin, S.-Y. Ho, et al. miRTarBase update 2014: an information resource for experimentally validated miRNA-target interactions. *Nucleic acids research*, 42(D1):D78–D85, 2014.

[8] R. Pesch and R. Zimmer. Cross-Species Conservation of Context-Specific Networks. *BMC System Biology*, 10(1):76, 2016.

[9] E. Rintala, P. Jouhten, M. Toivari, M. G. Wiebe, H. Maaheimo, M. Penttilä, and L. Ruohonen. Transcriptional responses of Saccharomyces cerevisiae to shift from respiratory and respirofermentative to fully fermentative metabolism. *Omics: a journal of integrative biology*, 15(7-8):461–476, 2011.

[10] S. B. Schawalder, M. Kabani, I. Howald, U. Choudhury, M. Werner, and D. Shore. Growth-regulated recruitment of the essential yeast ribosomal protein gene activator Ifh1. *Nature*, 432(7020):1058–1061, 2004.

[11] W. Sha, A. M. Martins, R. Laubenbacher, P. Mendes, and V. Shulaev. The genome-wide early temporal response of Saccharomyces cerevisiae to oxidative stress induced by cumene hydroperoxide. *PloS one*, 8(9):e74939, 2013.

[12] M. C. Teixeira, P. T. Monteiro, J. F. Guerreiro, J. P. Gonçalves, N. P. Mira, S. C. dos Santos, T. R. Cabrito, M. Palma, C. Costa, A. P. Francisco, et al. The YEASTRACT database: an upgraded information system for the analysis of gene and genomic transcription regulation in Saccharomyces cerevisiae. *Nucleic Acids Research*, pages D446–D451, 2013.
